# Prevalence of Elephant Endotheliotropic Herpesvirus (EEHV) in Wild and Captive Bornean Elephants (*Elephas maximus borneensis*) Sampled During Conservation Management Activities in Sabah, Malaysia

**DOI:** 10.3390/ani16193065

**Published:** 2026-09-29

**Authors:** Navaneetha Roopan, Nur Alwanie Maruji, Nur Nabila Sarkawi, Mei-Ho Lee, Thunyamas Guntawang, Worapong Kosaruk, Sharon S. Glaeser, Kidsadagon Pringproa, Symphorosa Sipangkui, Tom Hughes, Carlos R. Sanchez, Chatchote Thitaram

**Affiliations:** 1 Faculty of Veterinary Medicine, Chiang Mai University, Chiang Mai 50100, Thailand; thunyamas.g@cmu.ac.th (T.G.); worapong.kosa@cmu.ac.th (W.K.); kidsadagon.p@cmu.ac.th (K.P.); 2 Sabah Wildlife Department, Kota Kinabalu 88100, Sabah, Malaysia; nuralwanie.maruji@sabah.gov.my (N.A.M.); nabilasarkawi@gmail.com (N.N.S.); danguard78@gmail.com (S.S.); 3 Conservation Medicine, Sungai Buloh 47000, Selangor, Malaysia; mei-ho@conservationmedicine.org (M.-H.L.); tom@conservationmedicine.org (T.H.); 4 Center of Elephant, Wildlife and Companion Animals Research, Chiang Mai University, Chiang Mai 50100, Thailand; 5 Oregon Zoo, Portland, OR 97221, USA; sharon.glaeser@oregonzoo.org (S.S.G.); carlos.sanchez@oregonzoo.org (C.R.S.)

**Keywords:** Bornean elephant, Elephant Endotheliotropic Herpesvirus, prevalence, conservation management activities

## Abstract

Elephant Endotheliotropic Herpesvirus (EEHV) is a leading cause of fatal hemorrhagic disease in juvenile Asian elephants and represents a significant concern for elephant conservation. However, information on the epidemiology of EEHV in Bornean elephants (*Elephas maximus borneensis*) remains limited. This study investigated the prevalence and subtype distribution of EEHV in wild and captive Bornean elephants in Sabah, Malaysia, using samples collected during conservation management activities including rescue operations, translocation, GPS collaring, necropsies and routine veterinary examinations between 2009 and 2026. A total of 240 samples, comprising 100 whole-blood and 140 tissue samples from 110 elephants, were analyzed by conventional polymerase chain reaction (cPCR) targeting EEHV1 and EEHV4. EEHV DNA was detected in 6.4% (7/110) of elephants. Most positive cases were identified post-mortem, indicating a possible association of EEHV infection with mortality. EEHV1 was the only subtype detected, and sequence analysis showed close similarity to previously reported EEHV1A cases from Sabah. No EEHV4 was detected. Although no significant associations were observed between EEHV detection and population type, age group, sex, or sampling location, the detection of EEHV in both wild and captive elephants indicates that the virus is circulating in this endangered population. These findings provide baseline epidemiological data and support the need for continued surveillance and strengthened diagnostic capacity to inform EEHV management and conservation strategies in Sabah.

## Simple Summary

Elephant Endotheliotropic Herpesvirus (EEHV) is a major cause of sudden, often fatal disease in young Asian elephants and is an increasing concern for elephant conservation programs across Asia. However, little is known about this virus in the endangered Bornean elephant population in Sabah, Malaysia. Young elephants rescued from the wild are often brought into human care because of injury, orphaning, or conflict-related separation, making disease surveillance and veterinary health management especially important. In this study, blood and tissue samples collected from both wild and captive Bornean elephants between 2009 and 2026 were tested for EEHV using molecular diagnostic methods. A total of 110 elephants were included in the investigation. EEHV was detected in 6.4% of individuals, and retrospective examination of mortality cases identified EEHV in six elephants. This study provides the first baseline information on EEHV occurrence in Bornean elephants and highlights the importance of continued surveillance, rapid diagnosis, and long-term health monitoring to support conservation of this endangered subspecies.

## 1. Introduction

The Asian elephant (*Elephas maximus*) is classified as Endangered on the International Union for Conservation of Nature (IUCN) Red List of Threatened Species and is listed under Appendix I of the Convention on International Trade in Endangered Species of Wild Fauna and Flora (CITES) [1]. The Bornean elephant (*Elephas maximus borneensis*), which is endemic to the northern region of Borneo Island in Sabah, Malaysia, has been formally recognized by the IUCN as a subspecies of the Asian elephant [2]. It is a genetically distinct and evolutionarily significant lineage, with evidence supporting long-term isolation following the Pleistocene colonization of Borneo. Molecular and genomic studies indicate divergence from other populations approximately 170,000–300,000 years ago and refute a recent anthropogenic origin. Its restricted distribution and low genetic diversity underscore its status as a high-priority conservation unit [3,4,5]. In Sabah, the Bornean elephant is fully protected under Schedule 1 of the Wildlife Conservation Enactment 1997. The Bornean Elephant Action Plan 2020–2029 identifies habitat loss and fragmentation, mortality from various causes, limitations in species management, inadequate public awareness, and insufficient scientific knowledge as key threats to its long-term survival. Among these, elephant mortality remains a significant concern, with causes including human–elephant conflict (HEC) and retribution killing, hunting, poisoning, accidental deaths and infectious diseases [6]. Fatal diseases such as tuberculosis (TB) and Elephant Endotheliotropic Herpesvirus (EEHV) infection have been associated with morbidity and mortality in elephant populations in Malaysia [7,8,9] and worldwide [10]. Although the specific impact of TB and Elephant Endotheliotropic Herpesvirus Hemorrhagic Disease (EEHV-HD) on Bornean elephant population dynamics in Sabah has yet to be fully established, their potential contribution to mortality remains a conservation concern [6].

EEHVs are host-specific herpesviruses classified within the genus Proboscivirus in the subfamily Betaherpesvirinae of the family Orthoherpesviridae [11]. To date, eight EEHV genotypes—EEHV1 (subtypes 1A and 1B) and EEHV2–7—have been identified in African and Asian elephants [12,13,14]. Among these, EEHV1, EEHV4, and EEHV5 are ubiquitously detected in Asian elephants [15]. EEHV1A is the genotype most commonly associated with acute, highly fatal hemorrhagic disease in young Asian elephants, while EEHV4 and EEHV5 have also been implicated in co-infections and mortality [16,17]. Elephant Endotheliotropic Herpesvirus Hemorrhagic Disease (EEHV-HD) is the leading cause of death in juvenile Asian elephants in captivity worldwide, typically affecting individuals between 1 and 15 years of age [18,19]. Mortality is particularly high in elephants aged 1 to 8 years [20,21,22]. Clinically, EEHV-HD initially presents with clinical signs such as altered sleep patterns, lethargy, inappetence, pyrexia, generalized edema of the head and limbs, oral ulceration, and tongue cyanosis. The disease often progresses rapidly, causing widespread hemorrhage in major organs and death within 1–7 days in the absence of medical intervention [15,23,24,25].

Cases of EEHV-HD have been reported in both captive and wild elephants in Asian elephant range countries of India, Thailand, Laos, Cambodia, Myanmar, Nepal, Sumatra [26] and Malaysia [9]. Lesions consistent with EEHV infection have been documented in dead wild elephants, with subsequent laboratory confirmation of disease from necropsy samples [27,28,29]. The prevalence of EEHV has been investigated in ex situ populations in Western countries [18,30,31]. However, there remains a paucity of data on EEHV prevalence in wild elephant populations, and several studies have emphasized the need for systematic prevalence investigations to better understand disease epidemiology and pathogenesis [9,12,18,29,32,33,34,35].

In Sabah, current estimates indicate that the wild Bornean elephant population comprises approximately 1000 individuals [2], while an additional 29 elephants are living in human care under the jurisdiction of the Sabah Wildlife Department (SWD). Wild elephants are distributed across three Managed Elephant Ranges (MERs), namely Tabin, Lower Kinabatangan and Central Sabah [6]. The SWD continues to face increasing challenges due to the rescue of approximately three calves annually after being found separated from the herd, often due to injury, illness, or HEC (Sabah Wildlife Department, pers comms), with one published case of a calf being orphaned [36]. These rescued elephants fall within the age group most vulnerable to EEHV infection, and the cause of separation and the rescue process may induce psychological and physiological stress that could increase susceptibility to disease [37]. In 2016, the first fatal cases of EEHV were reported among rescued elephants in Sabah [9]. Since then, no published data have documented the status of EEHV in Bornean elephants, as the capacity for routine disease monitoring within the state remains under development. Therefore, it is critical to understand the potential impact of introducing new rescued elephants into rescue facilities on resident elephants and overall EEHV dynamics.

To the authors’ knowledge, this study is the first to assess the prevalence of EEHV infection in Bornean elephants in Sabah, Malaysia, using samples collected from both wild and captive individuals during conservation management activities. In addition, it aims to characterize the circulating EEHV genotypes within the population and conduct a retrospective analysis of EEHV-HD-associated mortality.

## 2. Materials and Methods

### 2.1. Study Populations and Sampling Locations

Between 2009 and 2026, 240 biological samples were collected from 110 Bornean elephants across Sabah, Malaysia (Table S1). The study population comprised wild (*n* = 58), captive (*n* = 37), and elephants of unrecorded origin (*n* = 15).

Demographic information was extracted from field reports and institutional records, including population type (wild/captive), geographical location for wild elephants, captive facility location and origin of birth (wild-born/captive-born) for captive elephants, age, and sex. Age classification was based on the following categories: calf (<1 year); juvenile (1–5 years); sub-adult (>5–15 years); adult (>15 years) [38].

#### 2.1.1. Wild Population

**Figure 1:**
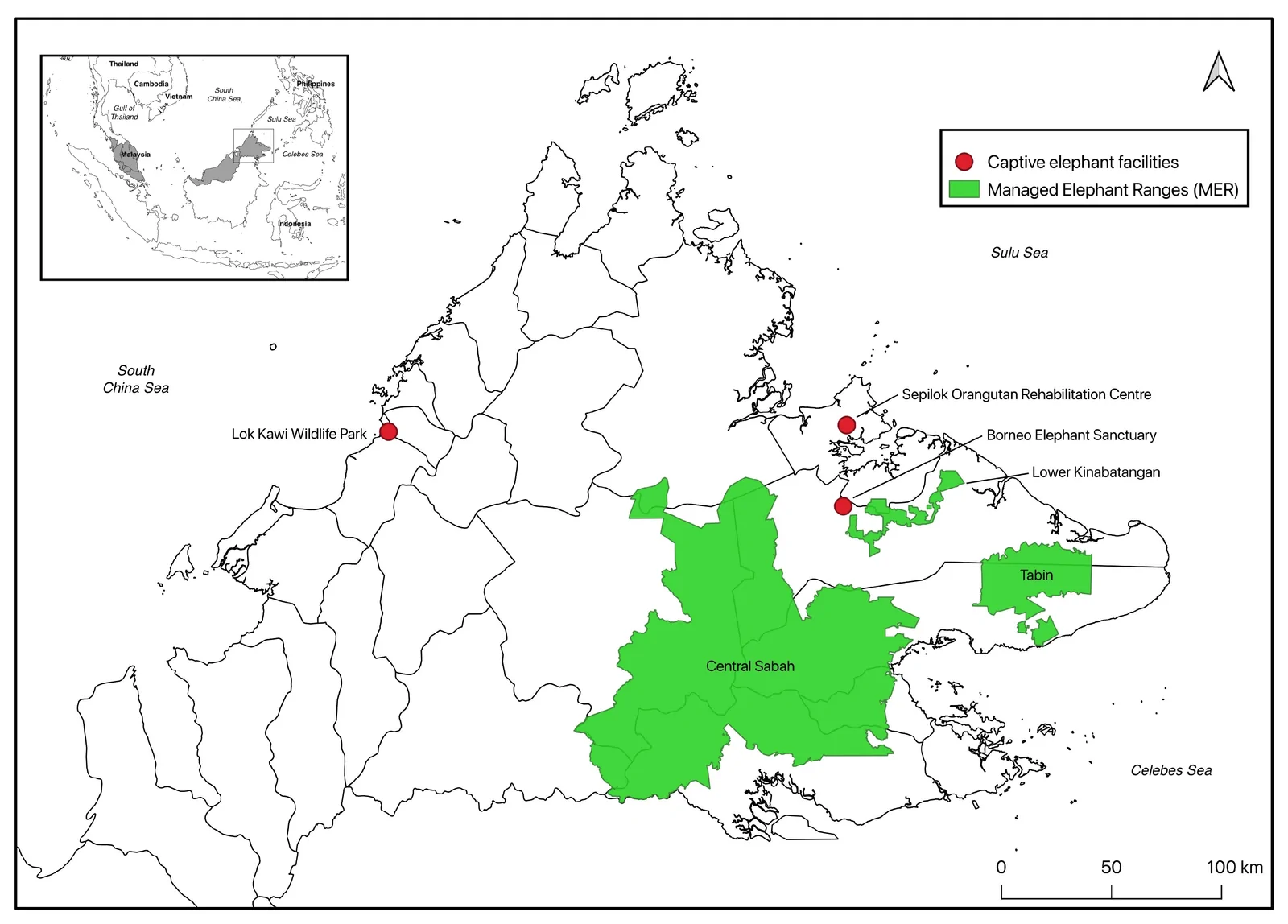
Geographic distribution of elephant populations and sampling sites included in the study in Sabah, Malaysia. Green areas represent the Managed Elephant Ranges (MERs), and red circles indicate captive elephant facilities where samples were collected. The inset shows the location of Sabah within Malaysia and Southeast Asia.

Wild elephants (*n* = 58) were sampled between 2012 and 2025. They originated from two of the three MERs in Sabah: Central Sabah (*n* = 36) and Lower Kinabatangan (*n* = 22) (Figure 1). No samples were allocated from Tabin for this study.

#### 2.1.2. Captive Population

Captive elephants (*n* = 37; sampled 2010–2026) were categorized by birth status as captive-born (*n* = 7) or wild-born (*n* = 30). For this study, wild-born captive elephants were defined as individuals rescued from the wild and managed under human care for at least one month prior to sampling. For these rescues, only the samples collected after one month in human care were used, so no individuals were included in both wild and captive cohorts. At sampling, elephants were housed at one of three captive facilities in Sabah: Borneo Elephant Sanctuary (BES; *n* = 6), Sepilok Orangutan Rehabilitation Centre (SORC; *n* = 11), or Lok Kawi Wildlife Park (LKWP; *n* = 20) (Figure 1).

The remaining 15 individuals could not be reliably assigned to a population type or location and were excluded from population-level analyses.

### 2.2. Sample Collection and Handling

Blood and tissue samples were collected as part of routine veterinary services and conservation management interventions by the SWD. The sampling location represents the MER for wild elephants and the captive facility for captive elephants.

Blood samples from wild elephants were collected opportunistically during state-mandated conservation management interventions, including rescue operations (with animals either remaining in the wild or being brought into human care, and sampling done in less than a month), translocations, and GPS collaring activities conducted since 2015. In contrast, blood samples from captive elephants were obtained during routine veterinary health assessments. A total of 5 mL of whole blood was collected from the auricular vein using 23G butterfly catheters or 21G to 23G, 1-to-1.5-inch hypodermic needles. All blood samples were immediately transferred into EDTA blood collection tubes.

Organ tissue samples were collected during forensic necropsies performed by the relevant authorities on deceased wild and captive elephants since 2013. The tissues collected and used in this study comprised heart, kidney, liver, lung, small intestine, spleen, and tongue (Table 1).

Immediately after collection, samples were stored at approximately 4 °C in portable coolers with ice packs before being transferred to a −20 °C freezer at the earliest opportunity. All specimens were subsequently transported to the Wildlife Health, Genetic and Forensic Laboratory (WHGFL), Kota Kinabalu, Sabah, for long-term storage at −80 °C.

### 2.3. EEHV Diagnostics

#### 2.3.1. Sample Processing (DNA Extraction)

DNA was extracted from biological samples (50 µL of whole blood or 25 mg of tissue) using the DNeasy^®^ Blood & Tissue Kit (Qiagen, Hilden, Germany) following the manufacturer’s protocol. DNA was then quantified using Qubit 4, and the concentrations were typically 8 × 10^−4^–112 × 10^−4^ µg/mL.

#### 2.3.2. EEHV cPCR Protocol

The DNA extracted from blood and tissue samples was used for conventional PCR (cPCR) for molecular screening. Three sets of primers were used in this study: EEHV1 TER-1 (218 bp) and EEHV4 TER-4 (310 bp), targeting the conserved terminase gene (U40) for subtype screening; and vGPCR (750 bp), targeting the hypervariable viral G-protein-coupled receptor 1 gene (U51) of EEHV1. While EEHV1 TER-1 and EEHV4 TER-4 were screened in all study samples in 2025–2026 to maximize diagnostic detection in field-derived DNA, the vGPCR system was applied to three samples in 2020 (W37) and 2021 (C01 and C21) (Table 2). The vGPCR target provides complementary value by yielding a longer, hypervariable sequence suitable for finer-scale strain discrimination and molecular epidemiological comparisons. DNA was amplified in 20 µL using Quick Taq HS DyeMix (Toyobo, Osaka, Japan) by adding 1×PCR master mix, 5 µM of primer mix and 2 µL of DNA template. PCR amplification for both EEHV1 and EEHV4 shared identical cycling conditions starting with initial denaturation at 94 °C for 2 min; 35 cycles of 94 °C for 30 s, annealing for 30 s (58 °C for EEHV1; 52 °C for EEHV4), and 72 °C for 40 s, followed by a final extension at 72 °C for 7 min. For vGPCR, the PCR cycling conditions were 95 °C for 2 min, then 45 cycles, with 1 cycle consisting of 95 °C for 40 s, 50 °C for 45 s, and 73 °C for 1 min, followed by a final step of 73 °C for 5 min. All PCRs were conducted using calibrated 96-well thermal cyclers (Veriti and SimpliAmp, Applied Biosystem, Waltham, MA, USA). Amplification was visualized using 1.2% agarose gel prepared in 0.5×TAE buffer, stained with 2.5 µL SYBR Safe, then placed on an LED transilluminator for visualization. DNA bands were cut and extracted from the gel using the QIAquick^®^ PCR & Gel Cleanup Kit (Qiagen, Hilden, Germany). DNA amplicons of the correct size were sequenced in both forward and reverse directions by Sanger sequencing at a commercial laboratory (Apical Scientific Sdn. Bhd., Seri Kembangan, Malaysia).

### 2.4. Phylogenetic Analysis

The sequences from the DNA sequencing were trimmed at both ends to remove primer sequences and low-quality sequenced base pairs. Each consensus sequence was aligned using the Geneious Alignment tool of the Geneious Prime 2026.1.1 software (Auckland, New Zealand) to obtain the longest possible sequence of each PCR product. The aligned sequences were checked against the National Center for Biotechnology Information GenBank database, using the basic local alignment search tool for nucleotide (BLASTN 2.17.0) results.

All confirmed sequences, 14 reference sequences for the U40 Terminase gene, and reference sequences for the U51 vGPCR1 gene (Table S2A,B) from GenBank were aligned using the Geneious Prime 2026.1.1 Geneious Alignment. The aligned sequences were used to build a phylogenetic tree for each gene using the Geneious Prime 2026.1.1 Tamura-Nei model of nucleotide substitution, the neighbor-joining method, 1000 bootstrap replicates and bootstrap support threshold % = 0.

### 2.5. Statistical Analysis

Statistical analyses were performed in R version 4.5.1. Analyses were conducted at both animal and sample levels. At the animal level, an elephant was classified as EEHV-positive when EEHV DNA was detected in at least one sample type from that individual. At the sample level, each submitted specimen was treated as a separate observation to describe detection patterns by sample type.

Descriptive statistics were used to summarize population type, sampling location, birth status, sex, age groups, and sample type availability. Elephants with missing information for a given variable were excluded only from analyses involving that variable, resulting in variable-specific denominators. Overall and group-specific EEHV detection prevalence were calculated with exact binomial 95% confidence intervals. Associations between EEHV detection at the animal level and categorical variables were assessed using Fisher’s exact test. The test with simulated *p*-values was used for variables with more than two categories. Odds ratios and 95% confidence intervals were reported only for two-level comparisons.

Sample-level EEHV detection was summarized by sample type with exact binomial 95% confidence intervals. Statistical significance was set at α = 0.05.

## 3. Results

### 3.1. Prevalence and Distribution of EEHV

Molecular screening by either the TER or vGPCR system detected EEHV1 in 6.4% (7/110) of the Bornean elephants. Most positive detections were from post-mortem cases, accounting for 85.7% (6/7) of all infections. In contrast, only one elephant was identified as having a subclinical infection based on blood sample analysis (14.3%; 1/7) at the time of sampling.

EEHV prevalence varied between captive and wild populations. A total of 8.6% (5/58) of wild elephants tested positive, whereas 5.4% (2/37) of captive elephants tested positive. No EEHV was detected among individuals of unrecorded origins (0/15). Among wild elephants, all EEHV-positive cases were from dead animals (16.7%; 5/30), while no EEHV-positive cases were detected among live animals (0/28). Among captive elephants, EEHV was detected in 25.0% (1/4) of dead animals and 3.0% (1/33) of live animals.

#### 3.1.1. Location Distribution of EEHV Infection

Within the wild cohort (*n* = 58), EEHV-positive cases were detected in both MERs sampled, with prevalences of 11.1% (4/36) in Central Sabah and 4.5% (1/22) in Lower Kinabatangan. Within the captive cohort (*n* = 37), no positives cases were detected at BES (0/6) or SORC (0/11), whereas a prevalence of 10% (2/20) was detected at LKWP. Of the two EEHV-positive individuals at LKWP, one was a wild-born elephant (C01) that tested positive in a blood sample, while the other was a captive-born elephant (C21) that tested positive in both blood and tissue samples. No statistically significant associations were detected between animal-level EEHV1 detection and the evaluated variables: population type (*p* = 0.702), geographical location of wild elephants (*p* = 0.640), birth status among captive elephants (*p* = 0.347) or sampling location (*p* = 0.853) (Table 3). Full Fisher’s exact test results, including odds ratios where applicable and 95% confidence intervals, are provided in Appendix A, Table A1.

#### 3.1.2. Age and Sex Distribution of EEHV Infection

EEHV-positive individuals were detected across multiple age groups, with the highest proportion observed among juvenile elephants aged 1–5 years. However, no statistically significant association was detected between animal-level EEHV1 detection and age group (*p* = 0.434). Of the 23 juveniles sampled, four individuals (17.4%) tested positive for EEHV. Subadult elephants showed a positivity rate of 13.3% (2/15), while only one adult elephant tested positive (4.2%; 1/24). No EEHV-positive individuals were detected among calves (0/12) or among elephants with unrecorded age information (0/36).

Based on sex distribution, a higher proportion of positive detections occurred in males, although no statistically significant association was identified (*p* > 0.999). Five of 64 male elephants (7.8%) tested positive for EEHV, compared to two of 38 female elephants (5.3%). No EEHV-positive individuals were identified among elephants of undetermined sex (0/8) (Table 3).

### 3.2. Characterization of EEHV Subtypes

**Figure 2:**
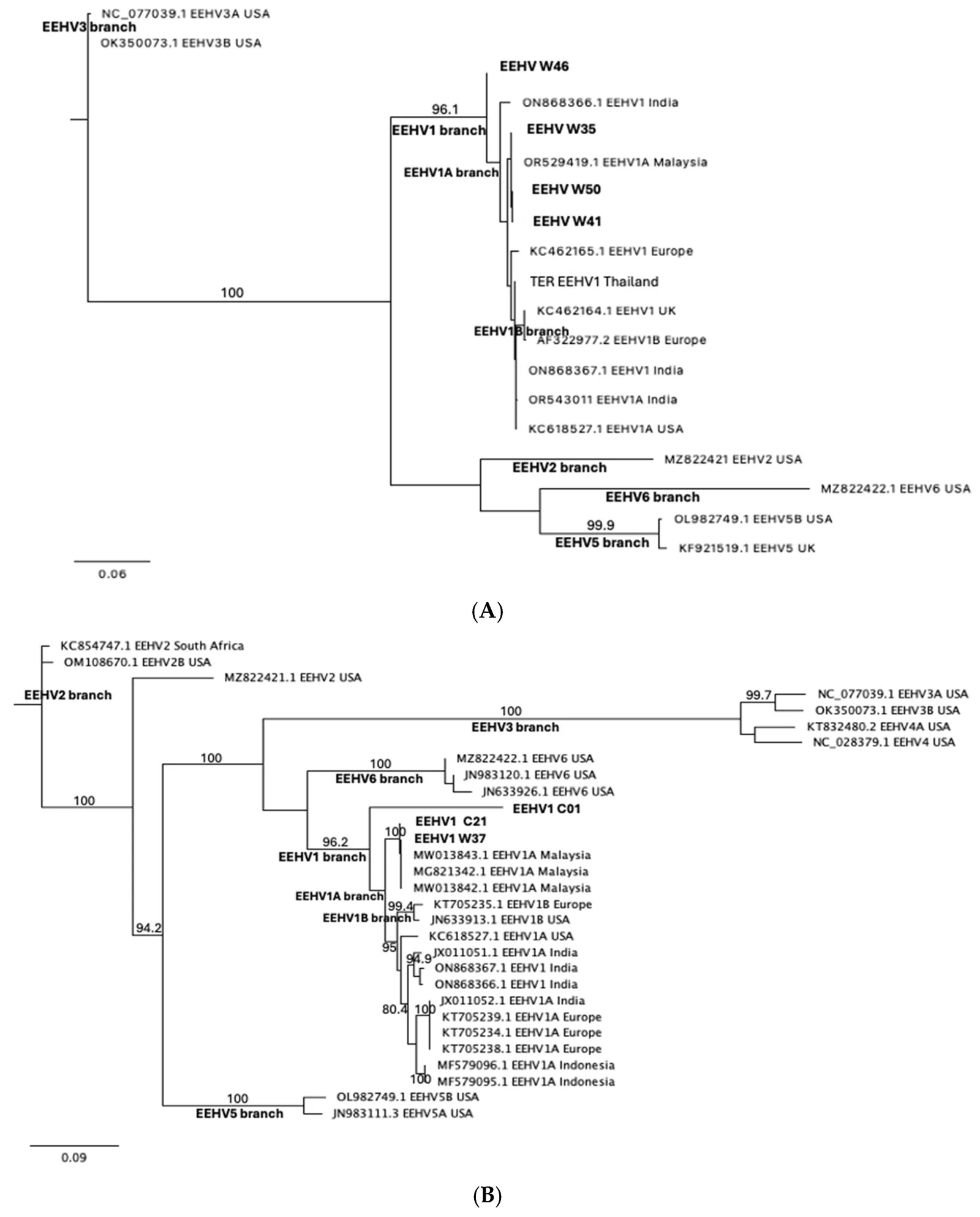
(**A**) A phylogenetic tree of the terminase U40 gene built using the neighbor-joining Tamura–Nei method with 1000 bootstrap replications. The bootstrap values are indicated at each branch. Bold font indicates positive samples (W35, W41, W46, W50) in this study and branch labels of the different subtypes. Only branch values > 80 are shown. (**B**) A phylogenetic tree of the vGPCR U51 gene built using the neighbor-joining Tamura–Nei method with 1000 bootstrap replications. The bootstrap values are indicated at each branch. Bold font indicates positive samples (C01, C21, W37) in this study and branch labels of the different subtypes. Only branch values > 80 are shown.

Molecular characterization revealed that EEHV1 was the only subtype detected in all positive cases. EEHV4 was not detected in any of the tested samples. The search against GenBank showed six PCR products from the TER 1 system (W37, C21, W35, W41, W46, W50) with 97–100% sequence identity to EEHV1A terminase U40 gene (Figure 2A). The search against GenBank showed all three PCR products from the vGPCR system (W37, C01, C21) with 96–100% sequence identity to EEHV1A vGPCR U51 gene (Figure 2B).

### 3.3. Comparative Analysis of Sample Types

EEHV detection rates varied by sample matrix, with tissue specimens obtained through necropsy yielding a higher detection rate of 6.4% (9/140), compared to 2.0% (2/100) for whole-blood samples. In total, seven positive tissue samples were obtained from five elephants (W35, W37, W41, W46 and W50); one positive blood sample was obtained from one elephant (C01); and one positive blood sample plus two positive tissue samples were detected in one elephant (C21), giving 11 positive samples from seven individuals. Among the positive individuals, the liver and heart each yielded the highest number of detections (*n* = 3), followed by the spleen (*n* = 2), blood (*n* = 2), and lung (*n* = 1). In contrast, all samples from the kidney, small intestine, and tongue were negative (Table 4).

## 4. Discussion

### 4.1. Prevalence and Distribution of EEHV in Sabah, Malaysia

This study provides the first systematic investigation of EEHV infection in both wild and captive Bornean elephants sampled during conservation management activities in Sabah, Malaysia, over a 17-year period (2009–2026). The overall animal-level prevalence of 6.4% (7/110) is consistent with EEHV circulation. This relatively low detection rate is consistent with PCR-based surveillance studies reported in other Asian elephant range countries [35,41]. This could be due to sample type, viral shedding dynamics, assay sensitivity, and possible regional differences in infection patterns.

Reports of EEHV in Malaysia remain limited and have focused mainly on captive elephants. The first confirmed cases of EEHV-HD was reported in wild-rescued elephants at a captive facility in Sabah [9], whereas subclinical EEHV infections were reported in a captive facility in Peninsular Malaysia [7]. In contrast, the present study included both wild and captive individuals and detected EEHV DNA across multiple sample types from the Bornean elephant population. Notably, more than 80% of all positive cases were identified post-mortem, underscoring the pathogenic potential of EEHV in this cohort, and indicating that fatal cases first reported in 2016 were not isolated events, but rather part of an ongoing threat to the population.

Among wild Bornean elephants, EEHV1 infection was detected in over 15% of dead individuals, suggesting a possible association with mortality, although gross pathological evidence of EEHV-HD was not available. A comparable pattern was reported in Thailand [27], where EEHV was detected in 30% (3/10) of dead wild elephants, including EEHV1 and EEHV4. Given the highly fragmented and isolated nature of Bornean elephant populations [42], this finding highlights the conservation concern posed by EEHV to this endangered subspecies. One possible explanation is that limited pathogen exposure may influence immunity within isolated populations [43], raising further concern about the immune status of wild elephants in Sabah, including rescue elephants coming into human care. However, serological studies are needed to evaluate this hypothesis.

No significant association was detected between sampling location and EEHV detection. Negative PCR results in captive elephants may reflect the absence of active shedding or subclinical viraemia at the time of sampling, but they do not exclude latent infection or intermittent viral circulation within the population [18,41,44]. No robust conclusion can be drawn from the variation in prevalence detected between Central Sabah MER (11.1%) and Lower Kinabatangan MER (4.5%). Given the small number of EEHV-positive cases and the uneven, opportunistic sampling between ranges, the observed difference should be interpreted cautiously. Further targeted surveillance with more balanced sampling across MERs is required to assess whether EEHV prevalence truly varies among populations.

### 4.2. Age and Sex Distribution of EEHV Infection in Sabah, Malaysia

In this study, younger elephants, particularly juveniles, were more frequently associated with EEHV infection than other age groups. This is consistent with the findings from studies in Thailand, which showed EEHV is more likely to be detected in juvenile elephants than in other age groups [27,34]. A higher occurrence of EEHV infection was also observed in males than in females in this study, which is in agreement with reports from Thailand [27,32]. However, the association of EEHV detection with either age group or sex was not significant, consistent with findings from Thailand [27].

### 4.3. Characterization of EEHV Subtypes of Bornean Elephants in Sabah

Although EEHV4 was not detected in any of the samples tested in this study, previous investigations in captive elephants in Switzerland, Peninsular Malaysia, and Thailand have reported EEHV4 infection in Asian elephants [7,27,30]. The absence of EEHV4 detection in the present study suggests that this subtype is less commonly circulating in the Bornean elephant population or occurs at a relatively low prevalence, as has been reported in other Asian elephant populations [12,22,34]. However, the limited sample size and opportunistic sampling design may have reduced the likelihood of detecting infection or intermittent EEHV4 shedding. Additional surveillance with broader sampling coverage may be required to confirm its presence or absence in this population. More extensive sampling may be required for the detection of EEHV4.

Phylogenetic analysis showed that the positive samples in this study were most closely related to previously reported EEHV cases from Sabah [9], which were identified as EEHV1A, followed by other EEHV1 cases reported elsewhere (Figure 2). One sample, C01, was found to be EEHV1 positive by the vGPCR gene cPCR conducted in 2021, but not by the terminase gene cPCR performed in 2026. This discrepancy may reflect a low viral DNA content in the sample or degradation of the extracted viral DNA used for both cPCRs in 2021 and 2026. Despite this discrepancy, the original positive vGPCR result was retained and C01 was included in the prevalence analysis.

The use of two distinct PCR screening systems in this study reflects the temporal development of diagnostic capacity in Sabah. The vGPCR system was initially applied in 2020–2021 due to resource limitations at WHGFL at the time of testing. Subsequently, the TER system (TER-1 and TER-4) was selected for state-wide screening in 2025–2026 because its shorter target amplicons (218 bp and 310 bp) offer higher analytical sensitivity when processing opportunistically collected field and post-mortem samples that may have suffered DNA degradation. While short terminase fragments are ideal for diagnostic detection and subtype identification, hypervariable loci like vGPCR provide complementary value for finer-scale strain discrimination and historical alignment.

The cPCR assays and subsequent phylogenetic analyses conducted in this study were primarily designed for diagnostic screening and subtype identification (e.g., confirming EEHV1A vs. EEHV4) rather than assessing fine-scale genetic variation among individual elephants or localized populations. Short genomic targets, such as the terminase (TER-1, 218 bp) and vGPCR (750 bp) loci, were prioritized because opportunistically collected field and necropsy samples often suffer from degraded or low-yield viral DNA due to tropical field conditions and transport delays. While these conserved fragments are highly effective for robust strain typing and initial phylogenetic confirmation, they lack sufficient nucleotide variation across the viral genome to resolve subtle intra-subtype divergence or transmission networks. To bridge the gap between preliminary subtype identification and full genomic characterization, intermediate sequencing strategies—e.g., those targeting complete, highly variable genes, full-length vGPCR (U51) and DNA polymerase (U38), or multi-locus gene sequencing panelsoffer a practical ‘go-between’. These informative loci provide greater sequence variation for strain discrimination and molecular epidemiological comparisons while remaining feasible for field-derived samples. Ultimately, where high-quality viral DNA and sufficient resources are available, whole-genome sequencing (WGS) will represent a major advancement in understanding genome-wide diversity, recombination events, and evolutionary history of EEHV in Bornean elephants.

### 4.4. Comparative Analysis of Sample Types of Bornean Elephants in Sabah

In the present study, EEHV DNA was most frequently detected in liver and heart tissues, consistent with previous reports demonstrating marked viral tropism and hemorrhagic lesions in these organs [13,24,45]. These findings support prioritization of heart, liver and spleen tissues for molecular diagnostics during necropsy investigations of suspected EEHV-HD cases. However, blood and lung tissue should not be neglected, given the pathogenesis of EEHV infection, which involves viremia and viral tropism in the lungs [16,20,24,46]. During necropsy, priority should be given to collecting these tissues, particularly in field settings where time and sample-storage capacity can be limited.

### 4.5. Conservation Implications and Management Recommendations

In the present study, EEHV DNA was detected in tissue samples of five wild Bornean elephants, raising concern about EEHV-related morbidity and herd immunity in wild elephants in Sabah. While PCR remains the gold standard for detecting EEHV shedding, diagnosing active infection, and monitoring disease progression or treatment responses [27,30,35], serological assays provide crucial insights into herd immunity [18,32] and may be more suitable for estimating population-level prevalence. Serological studies are needed to evaluate EEHV exposure and antibody levels in these fragmented populations, and to assess whether reduced opportunities for early infection may limit the development of protective herd immunity and increase vulnerability to EEHV-associated disease [18]. Furthermore, establishing baseline seroprevalence data in wild elephants would enable comparisons of EEHV exposure across MERs and could contribute to evidence-based conservation planning for elephant translocations, rehabilitation and release, and habitat connectivity priorities.

The elephant rescue situation in Sabah presents a unique challenge, primarily due to the majority of the rescued elephants belonging to high-risk age groups susceptible to EEHV. A significant proportion of rescued wild elephants are of nursing age and may be at risk of inadequate transfer of passive immunity, potentially increasing susceptibility to EEHV infection. It is critical to establish a baseline serological profile for resident captive individuals and assess the serostatus of newly rescued calves and juveniles. Coupled with real-time data on shedding activities, these efforts can significantly enhance the management strategies employed during the integration of at-risk elephants [47]. This comprehensive approach will facilitate informed disease management of these vulnerable populations.

### 4.6. Study Limitations

Several limitations should be considered when interpreting the findings of this study. Prevalence estimates reported here should be interpreted within the context of opportunistic sampling and may not represent true population prevalence in Bornean elephants throughout Sabah. Sampling was uneven across age groups, sexes, population type, and sampling locations, and the relatively small number of EEHV-positive individuals (*n* = 7) limited statistical power to detect significant associations between EEHV detection and demographic or management-related variables.

Compromised sample quality may also have contributed to the low rate of EEHV DNA detection, particularly in samples collected during field necropsies. The tropical climate of northern Borneo presents considerable challenges for field necropsy and post-mortem sample preservation. Prolonged exposure to high ambient temperatures, humidity, scavengers, insects, and direct sunlight accelerates autolysis and microbial degradation, thereby compromising tissue integrity [48,49,50] and nucleic acid stability and reducing molecular detection sensitivity [51].

Elephant carcasses in remote forested areas are often discovered at advanced stages of decomposition (A. Zachariah, pers. comms), which limits the quality of samples available for diagnostic evaluation and constrains gross pathological assessment. Because most tissues in this study were collected during field necropsies, degradation of viral DNA cannot be excluded. In addition, samples collected in eastern Sabah had to be transported to WHGFL on the west coast, which is distant from both the wild elephant ranges and the captive elephant facilities in eastern Sabah. These logistical constraints prolong time and may have reduced detection sensitivity during sample collection, handling, and transportation [52], thereby contributing to the low detection rate observed in this study and potentially underestimating the true prevalence of infection in the studied population. Collectively, these challenges highlight the need for standardized protocols for collection of samples from wild elephants, continued technical support and regular training for field personnel, rapid sample transfer to diagnostic laboratories, and cold-chain preservation systems to improve sample quality and diagnostic accuracy for future EEHV investigations in Sabah.

Despite these limitations, this study demonstrates that samples stored at −80 °C remain valuable for retrospective EEHV investigations in range countries where immediate laboratory access is limited, supporting the feasibility of future prevalence studies using archived material.

## 5. Conclusions

This study provides the first baseline epidemiological evidence of EEHV occurrence in Bornean elephants and highlights important considerations for disease surveillance in the management of a species of high conservation priority. However, the prevalence estimates reported here should be interpreted within the context of opportunistic conservation management sampling and should not be considered representative of the entire Bornean elephant population. EEHV was detected in both captive and free-ranging elephants in Sabah, with most positive cases identified post-mortem, indicating that the virus is actively circulating and may contribute to mortality in this endangered population. The detection of EEHV1A and the absence of EEHV4 in the sampled elephants suggest subtype-specific patterns that warrant further investigation. The findings emphasize the need for continued monitoring of EEHV in both wild and captive elephant populations in Sabah. Future studies should prioritize seroprevalence screening to better assess herd immunity and exposure patterns, particularly in juvenile elephants housed in captive facilities.

## Figures and Tables

**Table 1:** Details of samples obtained from veterinary services and necropsies during conservation management activities between 2009 and 2026 by location.

	Wild		Captive			Location Unknown
Samples for PCR	Lower Kinabatangan	Central Sabah	BES	SORC	LKWP	
Blood	11	19	9	17	29	15
Heart	13	13	1	1	2	NA
Liver	8	14	1	1	2	NA
Lung	13	12	1	NA	1	NA
Kidney	8	11	1	NA	NA	NA
Small intestine	8	3	NA	NA	NA	NA
Spleen	10	10	1	1	1	NA
Tongue	1	2	NA	NA	NA	NA

BES = Borneo Elephant Sanctuary; SORC = Sepilok Orangutan Rehabilitation Centre; LKWP = Lok Kawi Wildlife Park; NA = Sample not available.

**Table 2:** cPCR primers used in this study.

Target Gene	Primer Sequences (5′–3′)	Product Length (bp)	Reference
Terminase	EEHV1 TER-1 F: TTTATGCTCCTCTCCTCTGTGC EEHV1 TER-1 R: TATGCAGCACGGTTAACACG	218	[39]
Terminase	EEHV4 TER-4 F: GTGCTGTAGCGGATCATGTC EEHV4 TER-4 R: CGTGCAACACGAGCACGCAAAG	310	[23]
Viral G protein-coupled receptor (vGPCR)	Round 1: LGH7506 F: GATTGTGAACGCTGTATGCT LGH4963B R: GACTTTCTTCGTAGCCCTCGTCTT Round 2: LGH7506 F: GATTGTGAACGCTGTATGCT LGH5200A R: CGTGATACGCTTCAAAACATACA	Round 1: 910 Round 2: 750	[9,40]

**Table 3:** Demographic distribution of EEHV in Bornean elephants.

Categories	Tested Individuals	EEHV-Positive Individuals	Prevalence (95% CI)
**Age**			
Calf (0–1 year)	12	0	0.0% (0.0–26.5)
Juvenile (>1–5 years)	23	4	17.4% (5.0–38.8)
Subadult (>5–15 years)	15	2	13.3% (1.7–40.5)
Adult (>15 years)	24	1	4.2% (0.1–21.1)
NA	36	0	0.0% (0.0–9.7)
**Sex**			
Male	64	5	7.8% (2.6–17.3)
Female	38	2	5.3% (0.6–17.7)
NA	8	0	0.0% (0.0–36.9)
**Captive Facility**			
Borneo Elephant Sanctuary (BES)	6	0	0.0% (0.0–45.9)
Sepilok Orangutan Rehabilitation Centre (SORC)	11	0	0.0% (0.0–28.5)
Lok Kawi Wildlife Park (LKWP)	20	2	10.0% (1.2–31.7)
**MERs**			
Central Sabah	36	4	11.1% (3.1–26.1)
Lower Kinabatangan	22	1	4.5% (0.1–22.8)
Tabin	0	NA	NA
**Birth Status of Captive**			
Captive-born	7	1	14.3% (0.4–57.9)
Wild-born	30	1	3.3% (0.1–17.2)

NA = Data not available.

**Table 4:** Demography of EEHV-positive individuals.

No.	Research ID	Age	Sex	Population Type	Birth Status	Sampling Location	Sample Type							
							Blood	Heart	Liver	Lung	Kidney	Small Intestine	Spleen	Tongue
1	C01	J	M	Captive	wild-born	LKWP	+	NA	NA	NA	NA	NA	NA	NA
2	W35	J	F	Wild		CS	NA	−	−	+	−	NA	−	−
3	C21	J	M	Captive	captive-born	LKWP	+	+	+	NA	NA	NA	+	NA
4	W37	SA	M	Wild		CS	NA	−	+	−	−	NA	+	NA
5	W41	A	F	Wild		CS	NA	NA	+	NA	−	NA	NA	NA
6	W46	J	M	Wild		LK	NA	+	NA	−	−	−	−	NA
7	W50	SA	M	Wild		CS	NA	+	−	NA	−	NA	NA	NA

Age: J = Juvenile; SA = Sub-adult; A = Adult. Sex: M = Male; F = Female. Sample location: LKWP = Lok Kawi Wildlife Park; CS = Central Sentral; LK = Lower Kinabatangan. (+) = EEHV-positive samples; (−) = EEHV-negative samples; NA = Sample not available.

## Data Availability

The original contributions presented in this study are included in the article/Supplementary Materials. Further inquiries can be directed to the corresponding authors.

## Supplementary Materials

The following supporting information can be downloaded at: https://www.mdpi.com/article/10.3390/ani16193065/s1, Table S1: Sample ID and origin of the 110 Bornean elephants included in the study; Table S2A: Sequences used for the TER U40 gene phylogenetic analysis; Table S2B. Sequences used for the vGPCR1 U51 gene phylogenetic analysis.

## Appendix A

**Table A1:** Fisher’s exact test results.

Variable	Test	Included_n	*p*_Value	Odds_Ratio	Conf_Low	Conf_High	Note	*p*_Value_fmt
**Age class**	Fisher’s exact test with simulated *p*-value	74	0.3124687531246880	NA	NA	NA	A single odds ratio is not applicable for variables with >2 categories	0.312
**Sex**	Fisher’s exact test	102	1	1.519	0.234	16.756	–	>0.999
**Sampling** **location**	Fisher’s exact test with simulated *p*-value	95	0.8316168383161680	NA	NA	NA	A single odds ratio is not applicable for variables with >2 categories	0.832
**Birth status**	Fisher’s exact test	37	0.3468468468468470	0.22	0.003	18.927	–	0.347
**Population type**	Fisher’s exact test	95	0.7016694449870010	1.643	0.252	18.151	–	0.702
**Wild MER**	Fisher’s exact test	58	0.6400483968542040	0.387	0.007	4.27	–	0.640

NA = Not applicable.
