# Supplementary material for: Prevalence of Elephant Endotheliotropic Herpesvirus (EEHV) in Wild and Captive Bornean Elephants (Elephas maximus borneensis) Sampled During Conservation Management Activities in Sabah, Malaysia
Source: Animals (Basel). 2026 Sep 29;16(19):3065. doi: 10.3390/ani16193065 (PMC13663577; doi:10.3390/ani16193065)
Supplement: Supplementary file 1 [file animals-16-03065-s001.zip › Supplementary Table S1.pdf]

Table S1 Sample ID and origin of the 110 Bornean elephants included in the study

No.	Research ID	Sex	Age	Captive v. Wild	Birth Status for Captive	Captive Facility or MERs	Sample Type
1	W01	Male	Unrecorded	wild	n/a	CS	Blood
2	W02	Female	Unrecorded	wild	n/a	LK	Blood
3	W03	Female	Unrecorded	wild	n/a	CS	Blood
4	W04	Male	Unrecorded	wild	n/a	CS	Blood
5	W05	Male	Unrecorded	wild	n/a	CS	Blood
6	W06	Male	Unrecorded	wild	n/a	CS	Blood
7	W07	Female	Unrecorded	wild	n/a	CS	Blood
8	W08	Female	Unrecorded	wild	n/a	CS	Blood
9	W09	Male	Unrecorded	wild	n/a	LK	Blood
10	W10	Male	Unrecorded	wild	n/a	CS	Blood
11	W11	Male	Unrecorded	wild	n/a	CS	Blood
12	W12	Female	Unrecorded	wild	n/a	CS	Blood
13	W13	Female	Unrecorded	wild	n/a	CS	Blood
14	W14	Male	Unrecorded	wild	n/a	LK	Blood
15	W15	Male	Unrecorded	Wild	n/a	CS	Blood
16	W16	Male	Unrecorded	wild	n/a	CS	Blood
17	W17	Male	Unrecorded	wild	n/a	LK	Blood
18	W18	Male	Unrecorded	wild	n/a	LK	Blood
19	W19	Unrecorded	Unrecorded	wild	n/a	CS	Blood
20	W20	Male	Unrecorded	wild	n/a	CS	Blood
21	W21	Female	Unrecorded	wild	n/a	LK	Blood
**22**	**C01**	**Male**	**Juvenile**	**captive**	**wild-born**	**LKWP**	**Blood 1, Blood 2***
23	C02	Male	Juvenile	captive	wild-born	SORC	Blood 1, Blood 2, Blood 3

No.	Research ID	Sex	Age	Captive v. Wild	Birth Status for Captive	Captive Facility or MERs	Sample Type
24	W22	Female	Adult	wild	n/a	LK	Blood, H, Lu, SI, Sp
25	W23	Male	Adult	wild	n/a	LK	Blood, H, K, Lu, SI, Sp
26	W24	Male	Adult	wild	n/a	LK	Blood
27	W25	Female	Unrecorded	wild	n/a	LK	Blood
28	U01	Unrecorded	Unrecorded	Unknown	n/a	n/a	Blood
29	U02	Female	Unrecorded	Unknown	n/a	n/a	Blood
30	U03	Male	Unrecorded	Unknown	n/a	n/a	Blood
31	C03	Male	Adult	captive	wild-born	LKWP	Blood
32	U04	Female	Unrecorded	Unknown	n/a	LK	Blood
33	U05	Female	Unrecorded	Unknown	n/a	n/a	Blood
34	U06	Male	Unrecorded	Unknown	n/a	n/a	Blood
35	U07	Female	Adult	Unknown	n/a	n/a	Blood
36	W26	Female	Unrecorded	wild	n/a	CS	Blood
37	U08	Female	Unrecorded	Unknown	n/a	n/a	Blood
38	U09	Female	Unrecorded	Unknown	n/a	n/a	Blood
39	C04	Male	Sub-adult	captive	captive-born	LKWP	Blood 1, Blood 2
40	C05	Male	Sub-adult	captive	captive-born	LKWP	Blood 1, Blood 2
41	W27	Female	Calf	wild	n/a	LK	Blood
42	C06	Male	Calf	captive	wild-born	LKWP	Blood, H, Li, Lu
43	U10	Unrecorded	Unrecorded	Unknown	n/a	n/a	Blood
44	U11	Unrecorded	Unrecorded	Unknown	n/a	n/a	Blood
45	U12	Unrecorded	Unrecorded	Unknown	n/a	n/a	Blood
46	U13	Unrecorded	Unrecorded	Unknown	n/a	n/a	Blood
47	W28	Male	Calf	Wild	n/a	CS	Blood
48	U14	Male	Juvenile	Unknown	n/a	n/a	Blood

No.	Research ID	Sex	Age	Captive v. Wild	Birth Status for Captive	Captive Facility or MERs	Sample Type
49	C07	Male	Sub-adult	captive	wild-born	LKWP	Blood 1, Blood 2
50	U15	Female	Adult	Unknown	n/a	n/a	Blood
51	C08	Male	Calf	Captive	wild-born	SORC	Blood
52	C09	Male	Juvenile	Captive	wild-born	SORC	Blood
53	C10	Male	Juvenile	Captive	wild-born	SORC	Blood
54	C11	Male	Juvenile	Captive	wild-born	SORC	Blood
55	C12	Male	Juvenile	Captive	wild-born	SORC	Blood
56	C13	Male	Calf	Captive	wild-born	BES	Blood
57	C14	Male	Sub-adult	Captive	wild-born	SORC	Blood 1, Blood 2
58	C15	Male	Sub-adult	Captive	wild-born	SORC	Blood 1, Blood 2
59	C16	Male	Calf	Captive	wild-born	BES	Blood
60	C17	Male	Adult	Captive	wild-born	BES	Blood 1, Blood 2
61	C18	Male	Adult	Captive	wild-born	BES	Blood 1, Blood 2
62	C19	Male	Sub-adult	Captive	wild-born	SORC	Blood 1, Blood 2
63	C20	Male	Calf	Captive	wild-born	BES	Blood 1, Blood 2
64	W29	Female	Adult	Wild	n/a	CS	Blood
65	W30	Female	Juvenile	wild	n/a	CS	H, Li, Lu, Sp
66	W31	Male	Juvenile	wild	n/a	CS	H, Lu
67	W32	Male	Juvenile	wild	n/a	CS	H, Li, Lu, Sp
68	W33	Female	Calf	wild	n/a	LK	H, Li, Lu, Sp
69	W34	Male	Calf	wild	n/a	LK	H, Li, Lu, Sp
**70**	**W35**	**Female**	**Juvenile**	**wild**	**n/a**	**CS**	**H, K, Li, Lu*, Sp, T**
**71**	**C21**	**Male**	**Juvenile**	**captive**	**Captive born**	LKWP	**Blood*, H*, Li*, Sp***
72	C22	Female	Adult	captive	wild-born	LKWP	Blood 1, Blood 2
73	C23	Female	Adult	captive	wild-born	LKWP	Blood 1, Blood 2

No.	Research ID	Sex	Age	Captive v. Wild	Birth Status for Captive	Captive Facility or MERs	Sample Type
74	C24	Male	Adult	captive	wild-born	LKWP	Blood 1, Blood 2
75	C25	Male	Adult	captive	wild-born	LKWP	Blood 1, Blood 2
76	C26	Male	Sub-adult	captive	wild-born	SORC	Blood 1, Blood 2
77	C27	Male	Adult	captive	wild-born	LKWP	Blood
78	C28	Female	Adult	captive	wild-born	LKWP	Blood
79	C29	Female	Sub-adult	captive	wild-born	LKWP	Blood
80	C30	Male	Sub-adult	captive	wild-born	LKWP	Blood
81	C31	Female	Sub-adult	captive	captive-born	LKWP	Blood
82	C32	Male	Sub-adult	captive	wild-born	LKWP	Blood
83	C33	Male	Juvenile	captive	wild-born	SORC	Blood, H, Li, Sp
84	C34	Male	Adult	captive	wild-born	BES	Blood, H, K, Li, Lu, Sp
85	W36	Female	Calf	wild	n/a	CS	Heart, K, Li, Lu, SI, Sp
**86**	**W37**	**Male**	**Sub-adult**	**wild**	n/a	**CS**	**H, K, Li*, Lu, Sp***
87	W38	Male	Sub-adult	wild	n/a	LK	H, K, Li, Lu, SI, Sp
88	W39	Male	Calf	wild	n/a	CS	H, K, Li, Lu, Sp
89	W40	Male	Adult	Wild	n/a	CS	K, Li
**90**	**W41**	**Female**	**Adult**	**Wild**	n/a	**CS**	**K, Li***
91	W42	Unrecorded	Juvenile	Wild	n/a	LK	H, K, Li, Lu
92	W43	Female	Adult	Wild	n/a	CS	Liver
93	W44	Unrecorded	Calf	Wild	n/a	CS	H, K, Li, Sp
94	W45	Female	Sub-adult	wild	n/a	CS	H, Lu
**95**	**W46**	**Male**	**Juvenile**	**wild**	n/a	**LK**	**H*, K, Lu, SI, Sp**
96	W47	Male	Calf	wild	n/a	LK	H, Lu, SI, Sp
97	W48	Female	Adult	wild	n/a	LK	H, K, Li, Lu, SI, Sp, T
98	W49	Male	Juvenile	wild	n/a	LK	H, K, Li, Lu, Sp

No.	Research ID	Sex	Age	Captive v. Wild	Birth Status for Captive	Captive Facility or MERs	Sample Type
**99**	**W50**	**Male**	**Sub-adult**	**wild**	n/a	**CS**	**H*, K, Li**
100	W51	Male	Juvenile	wild	n/a	LK	H, K, Li, Lu, SI
101	W52	Male	Juvenile	wild	n/a	LK	H, K, Li, Lu, SI
102	W53	Male	Juvenile	wild	n/a	CS	H, K, Li, Lu, SI, Sp
103	W54	Male	Adult	wild	n/a	CS	H, K, Li, Sp
104	W55	Female	Adult	wild	n/a	CS	K, Li, Lu, SI, Sp, T
105	W56	Female	Adult	wild	n/a	CS	H, Lu
106	W57	Female	Adult	wild	n/a	LK	H, Lu, Sp
107	W58	Female	Unrecorded	wild	n/a	CS	Blood
108	C35	Male	Juvenile	captive	captive-born	BES	Blood
109	C36	Female	Juvenile	captive	captive-born	LKWP	Blood
110	C37	Male	Juvenile	captive	captive-born	LKWP	Blood

n/a = Not applicable; BES = Borneo Elephant Sanctuary; SORC = Sepilok Orangutan Rehabilitation Centre; LKWP = Lok Kawi Wildlife Park; CS = Central Sabah, LK = Lower Kinabatangan; H = Heart; K = Kidney; Li = Liver; Lu = Lung; SI = Small intestine; Sp = Spleen; T = Tongue; * = EEHV-positive sample
