# Supplementary material for: Prevalence of Elephant Endotheliotropic Herpesvirus (EEHV) in Wild and Captive Bornean Elephants (Elephas maximus borneensis) Sampled During Conservation Management Activities in Sabah, Malaysia
Source: Animals (Basel). 2026 Sep 29;16(19):3065. doi: 10.3390/ani16193065 (PMC13663577; doi:10.3390/ani16193065)
Supplement: Supplementary file 1 [file animals-16-03065-s001.zip › Supplementary Table S2 (S2A & S2B).pdf]

Table S2A. Sequences used for the TER U40 gene phylogenetic analysis.

Accession number	Description	EEHV subtype
OL982749.1	Elephant endotheliotropic herpesvirus 5B isolate North American NAP58 Tucker (USA)	5B
KF921519.1	Proboscivirus elephantidbeta 5 strain Vijay (UK)	5
MZ822421.1	Elephant endotheliotropic herpesvirus 2 isolate NAP12 Kijana (USA)	2
MZ822422.1	Elephant endotheliotropic herpesvirus 6 isolate NAP35 Miss Bets (USA)	6
NC_077039.1	Elephant endotheliotropic herpesvirus 3A isolate Nyah NAP97 (USA)	3A
OK350073.1	Elephant endotheliotropic herpesvirus 3B isolate North American NAP96 MB3 (USA)	3B
OR543011.1	Elephant endotheliotropic herpesvirus 1A isolate Umesh (EP55) (Switzerland)	1A
NA	Elephant endotheliotropic herpesvirus 1A (Thailand)	1A
KC618527.1	Elephant endotheliotropic herpesvirus 1A isolate Kimba NAP23 (USA)	1A
ON868367.1	Elephantid betaherpesvirus 1 strain EEHV\Chattishgarh\India\E8 (India)	1
OR529419.1	Elephant endotheliotropic herpesvirus 1A isolate Jimbo BP01 (Malaysia)	1A
KC462165.1	Elephantid herpesvirus 1 strain Raman (UK)	1A
ON868366.1	Elephantid betaherpesvirus 1 strain EEHV\Ranchi\India\E2 (India)	1
KC462164.1	Elephantid herpesvirus 1 strain Emelia (UK)	1B
AF322977.2	Elephantid herpesvirus 1 (Germany)	1

NA = Not available

Table S2B. Sequences used for the vGPCR1 U51 gene phylogenetic analysis.

Accession number	Description	EEHV subtype
MZ822422.1	Elephant endotheliotropic herpesvirus 6 isolate NAP35 Miss Bets, complete genome (USA)	6
JN633926.1	Elephant endotheliotropic herpesvirus 6 NAP42 G protein-coupled receptor (U51) gene, partial cds (USA)	6
JN983120.1	Elephant endotheliotropic herpesvirus 6 isolate North American #NAP35 U51 gene, partial cds (USA)	6
JN983111.3	Elephant endotheliotropic herpesvirus 5A isolate North American #NAP50, partial genome (USA)	5A
OL982749.1	Elephant endotheliotropic herpesvirus 5B isolate North American NAP58 Tucker (USA)	5B
NC_028379.1	Elephant endotheliotropic herpesvirus 4 isolate North American NAP69 complete genome (USA)	4
KT832480.2	Elephant endotheliotropic herpesvirus 4A G protein-coupled receptor 1 (U51) gene, complete cds (USA)	4A
NC_077039.1	Elephant endotheliotropic herpesvirus 3A isolate Nyah NAP97 (USA)	3A
OK350073.1	Elephant endotheliotropic herpesvirus 3B isolate North American NAP96 MB3, complete genome	3B
KC854747.1	Elephant endotheliotropic herpesvirus 2 isolate South African 54FLNG G protein-coupled receptor 1 (U51) gene, partial cds (South Africa)	2
OM108670.1	Elephant endotheliotropic herpesvirus 2B isolate NAP107 G protein-coupled receptor 1 (U51) gene, partial cds	2B
MZ822421.1	Elephant endotheliotropic herpesvirus 2 isolate NAP12 Kijana (USA)	2

KC618527.1	Elephant endotheliotropic herpesvirus 1A isolate Kimba NAP23 (USA)	1A
ON868367.1	Elephantid betaherpesvirus 1 strain EEHV\Chattishgarh\India\E8 (India)	1
ON868366.1	Elephantid betaherpesvirus 1 strain EEHV\Ranchi\India\E2 (India)	1
JX011052.1	Elephant endotheliotropic herpesvirus 1A isolate Indian IP43 G protein-coupled receptor 1 (U51) gene, partial cds (India)	1A
JX011051.1	Elephant endotheliotropic herpesvirus 1A isolate Indian IP11 G protein-coupled receptor 1 (U51) gene, partial cds (India)	1A
MF579096.1	Elephant endotheliotropic herpesvirus 1A isolate Tangka SP02 G protein-coupled receptor 1 (U51) gene, partial cds (Indonesia)	1A
MF579095.1	Elephant endotheliotropic herpesvirus 1A isolate Namo SP01 G protein-coupled receptor 1 (U51) gene, partial cds (Indonesia)	1A
KT705234.1	Elephant endotheliotropic herpesvirus 1A isolate European EP14 G protein-coupled receptor 1 (U51) gene, partial cds (Germany)	1A
KT705235.1	Elephant endotheliotropic herpesvirus 1B isolate European EP18 G protein-coupled receptor 1 (U51) gene, partial cds (UK)	1B
JN633913.1	Elephant endotheliotropic herpesvirus 1B isolate NAP33 G protein-coupled receptor (U51) gene, partial cds (USA)	1B
KT705238.1	Elephant endotheliotropic herpesvirus 1A isolate European EP23 G protein-coupled receptor 1 (U51) gene, partial cds (Germany)	1A
KT705239.1	Elephant endotheliotropic herpesvirus 1A isolate European EP25 G protein-coupled receptor 1 (U51) gene, partial cds (Germany)	1A
MG821342.1	Elephant endotheliotropic herpesvirus 1A isolate Jimbo BP01 G protein-coupled receptor 1 (U51) gene, partial cds (Malaysia)	1A
MW013842.1	Elephant endotheliotropic herpesvirus 1A isolate Tuntan BP03 G protein-coupled receptor 1 (U51) gene, partial cds (Malaysia)	1A
MW013843.1	Elephant endotheliotropic herpesvirus 1A isolate Vinodh BP04 G protein-coupled receptor 1 (U51) gene, partial cds (Malaysia)	1A
